# The PHO signaling pathway directs lipid remodeling in *Cryptococcus neoformans* via DGTS synthase to recycle phosphate during phosphate deficiency

**DOI:** 10.1371/journal.pone.0212651

**Published:** 2019-02-21

**Authors:** Sophie Lev, Thusitha Rupasinghe, Desmarini Desmarini, Keren Kaufman-Francis, Tania Christine Sorrell, Ute Roessner, Julianne Teresa Djordjevic

**Affiliations:** 1 Centre for Infectious Diseases and Microbiology, Fungal Pathogenesis Group, Westmead Institute for Medical Research, Westmead, New South Wales, Australia; 2 Sydney Medical School-Westmead, University of Sydney at Westmead Hospital, Westmead, New South Wales, Australia; 3 Marie Bashir Institute for Infectious Diseases and Biosecurity, University of Sydney, Sydney, New South Wales, Australia; 4 Metabolomics Australia, School of Biosciences, The University of Melbourne, Melbourne, Victoria, Australia; Yonsei University, REPUBLIC OF KOREA

## Abstract

The phosphate sensing and acquisition (PHO) pathway of *Cryptococcus neoformans* is essential for growth in phosphate-limiting conditions and for dissemination of infection in a mouse model. Its key transcription factor, Pho4, regulates expression of genes controlling the acquisition of phosphate from both external and cellular sources. One such gene, *BTA1*, is highly up-regulated during phosphate starvation. Given that a significant proportion of cellular phosphate is incorporated into phospholipids, and that the Pho4-dependent *BTA1* gene encodes an enzyme predicted to catalyse production of a phosphorus-free betaine lipid, we investigated whether phospholipids provide an accessible reservoir of phosphate during phosphate deficiency. By comparing lipid profiles of phosphate-starved WT *C*. *neoformans*, *PHO4* (*pho4Δ*) and *BTA1* (*bta1Δ*) deletion mutants using thin layer chromatography and liquid chromatography mass spectrometry, we showed that phosphatidylcholine (PC) is substituted by the phosphorus-free betaine lipids diacylglyceryl-N,N,N-trimethylhomoserine (DGTS) and diacylgyceryl hydroxymethyl-N,N,N-trimethyl-beta-alanine (DGTA) in a Pho4- and Bta1-dependent manner, and that *BTA1* encodes a functional DGTS synthase. Synthesis of DGTA tightly correlated with that of DGTS, consistent with DGTS being the precursor of DGTA. Similar to *pho4Δ*, *bta1Δ* grew more slowly than WT in cell culture medium (RPMI) and was hypovirulent in a murine model of cryptococcosis. In contrast to *pho4Δ*, *bta1Δ* tolerated alkaline pH and disseminated to the brain. Our results demonstrate that Bta1-dependent substitution of PC by betaine lipids is tightly regulated in *C*. *neoformans* by the PHO pathway, to conserve phosphate and preserve membrane integrity and function. This phospholipid remodeling strategy may also contribute to cryptococcal virulence during host infection.

## Introduction

Phosphorus in the form of phosphate (PO_4_^3–^, P_i_) is essential for cellular growth and function. Microorganisms, including fungi, are exposed to fluctuating levels of extracellular phosphate, depending on their environmental niche. In order to maintain a stable intracellular phosphate concentration, they have evolved tightly regulated mechanisms to sense, take up, store and utilize phosphate. In fungi, this process is dependent on the phosphate sensing and acquisition (PHO) pathway. The core signaling cascade of the PHO pathway consists of the cyclin-dependent kinase (CDK)-cyclin complex, the CDK inhibitor and a transcriptional regulator(s). When intracellular phosphate is low, activation of the pathway occurs and triggers expression of effector genes that encode proteins involved in the acquisition of phosphate from external sources and enzymes involved in recycling phosphate from internal sources (reviewed in [1,2]).

The opportunistic fungal pathogen *Cryptococcus neoformans* causes life-threatening meningitis, primarily in immunocompromised individuals, and is a major cause of morbidity and mortality worldwide [3]. *C*. *neoformans* initially infects the lungs, from which it is transported via the circulatory system to the brain, causing meningo-encephalitis. Within each site of infection, *C*. *neoformans* must acquire sufficient phosphate to sustain its growth and tolerate host-derived stress. We previously reported that the PHO pathway in *C*. *neoformans* is essential for its virulence in a murine model [4], and that PHO pathway function has expanded to facilitate acquisition of nutrients other than phosphate [2,5].

The PHO signaling cascade in *C*. *neoformans* is comprised of the CDK Pho85, the cyclin Pho80, the CDK inhibitor Pho81, with Pho4 being the sole transcriptional regulator [4,5]. Phosphate deprivation activates the PHO pathway leading to Pho4-mediated induction of acid phosphatases [6], high-affinity phosphate transporters [7], vacuolar polyphosphate polymerase [7] and transporters of nutrients other than phosphate [2,5]. The PHO pathway in *C*. *neoformans* and in *Candida albicans* is also activated at alkaline pH [8], suggesting that these yeasts experience phosphate deprivation during infection of the host. This is presumably because some phosphate transporters are proton symporters and thus function optimally at acidic but not at alkaline pH. Consequently, the *PHO4* deletion mutants (*pho4Δ*) of both *C*. *neoformans* and *C*. *albicans* are hypovirulent in a mouse infection model [4,8].

The fungal PHO pathway responds to phosphate deprivation in several ways. These include enhancing the capacity of the cell to (1) break down complex sources of extracellular phosphate via the hypersecretion of phosphatases, (2) import free phosphate from the exterior via enhanced synthesis of phosphate transporters, (3) utilise phosphate from intracellular stores (vacuolar polyP) via enhanced production of exo- and endo-polyphosphatases and (4) substitute membrane phospholipids with phosphorus-free betaine lipids. Although the first 3 strategies have been studied in *C*. *neoformans* [4–7], nothing is known about whether this yeast pathogen utilizes PHO pathway-dependent phospholipid remodeling to conserve phosphate during growth and infection.

A mechanism of phosphate conservation which involves replacement of phospholipids with phosphorus-free betaine lipids has been reported in bacteria [9–12], plants [13,14], algae [15,16] and fungi [17,18]. Diacylgyceryl-N-trimethylhomoserine (DGTS) is the most abundant microbial betaine lipid followed by diacylglyceryl hydroxymethyl-N,N,N-trimethyl-beta-alanine (DGTA) and diacylglyceryl carboxyhydroxymethylcholine (DGCC). DGTS is also the most ubiquitous betaine lipid in nature [16,19]. Betaine lipids have a similar net charge and structure to phosphatidylcholine (PC). They are zwitterionic, with a positively charged trimethylammonium group and a negatively charged carboxyl group in their polar head group. However, their head group is attached to the diacylglycerol backbone by an ether bond rather than by a phosphodiester bond.

The synthesis of DGTS in bacteria from diacylglycerol (DAG) via a diacylglycerylhomoserine intermediate is a two-step process catalysed by BtaA and BtaB [20,21]. However, only a single enzyme containing both BtaA- and BtaB-like domains performs all steps in DGTS synthesis in the green algae *Chlamydomonas reinhardtii* [21]. Genome analyses has revealed that orthologs of CrBta1 are found in some, but not all, ascomycetous and basidiomycetous fungi [17,18,22]. The model ascomycete, *Saccharomycetes cerevisiae*, does not produce Bta1 and hence does not contain betaine lipids. However, replacing PC in *S*. *cerevisiae* with DGTS produced from heterologously-expressed *BTA1* (from *K*. *lactis)* had no effect on cellular function, consistent with DGTS being a structural mimic of PC [17]. Unlike *S*. *cerevisiae*, other non-pathogenic fungi, such as *Flammulina velutipes*, *Neurospora crassa* and *K*. *lactis*, can substitute phospholipids with DGTS during phosphate deprivation [17,18]. More recently, DGTS production was shown to be essential for virulence of the plant fungal pathogen, *Fusarium graminearum*, promoting its growth in the phosphate-deficient apoplast of maize [22]. We found that during phosphate starvation, a putative DGTS synthase-encoding gene (*BTA1)* is up-regulated >100-fold in *C*. *neoformans* by Pho4 [4,5]. However, it is not known whether *BTA1* encodes a functional DGTS synthase. Furthermore, the role of Pho4 in lipid remodeling and the contribution of Bta1 to cryptococcal membrane composition and virulence have never been investigated.

In the present study we perform comparative Liquid Chromatography Mass Spectrometry (LC-MS)-based lipid profiling to investigate whether *C*. *neoformans* utilizes PHO pathway-dependent phospholipid remodeling as a phosphate conservation strategy. We then assess whether Pho4-dependent *BTA1* is involved and whether the presence of a functional DGTS synthase contributes to cryptococcal virulence in animal models.

## Materials and methods

### Fungal strains, growth media and plasmids

Wild-type *C*. *neoformans* var. *grubii* strain H99 (serotype A, MATa) was used in this study. Creation and verification of the *PHO4* (CNAG_06751) deletion mutant was described in [4].

Routinely, the cells were grown on Sabourad Dextrose Agar (SDA) or in YPD broth (1% yeast extract, 2% peptone, and 2% dextrose). Minimal medium without phosphate (MM-Pi-: 29.4 mM KCl, 15 mM glucose, 10 mM MgSO_4_.7H_2_O, 13 mM glycine, 3 μM thiamine-HCl) and minimal medium with phosphate (MM-Pi+: 29.4mM KH_2_PO_4_, 15mM glucose, 10mM MgSO_4_.7H_2_O, 13 mM glycine, 3 μM thiamine-HCl) were used for phosphate-deficient and phosphate-replete conditions, respectively. The MM cultures were routinely incubated with shaking at 30°C.

For the experiments requiring different pH, RPMI/FBS was prepared by combining RPMI powder (Sigma), glutamine and FBS (10%) with MES (100 mM) used for pH 5.4 and HEPES (100 mM) used for pH 7.4. Otherwise, commercially available RPMI 1640 was supplemented with 10% FBS and glutamine. Routinely, the cells in RPMI were incubated without shaking at 37°C, 5% CO_2_ for the times indicated.

### Creation of *bta1Δ* and *bta1Δ+BTA1* strains

The *BTA1* (CNAG_02353) deletion mutant *bta1Δ* was created by homologous recombination of a deletion construct created by overlap PCR. The 5’ flank of the *BTA1* deletion construct was amplified using primers BTA1-5’new2-s and BTA1-5’CDS-a using WT H99 genomic DNA as a template. It included 299 bp upstream of the *BTA1* coding sequence and the first 482 bp of the *BTA1* coding region. The neomycin resistance cassette (Neo^r^) was amplified from pJAF [23] using the primer pair Neo-s and Neo-a. The 3’ flank included a 998 bp fragment downstream of the *BTA1* ORF and was PCR amplified using primers BTA1-3’s and BTA1-3’a. The three fragments were joined by overlap PCR, with Neo^r^ in place of the coding region, and the resulting product was cloned into the pCR2.1 TOPO vector using the TOPO TA cloning kit (Invitrogen). The plasmid was linearized with the restriction endonuclease, KpnI, and introduced into the fungal cells by biolistic transformation [24]. Neomycin-resistant transformants were screened by PCR amplification across the junctions of homologous recombination (**S1 Fig**).

To re-introduce the native *BTA1* gene into the *bta1Δ* strain we used the plasmid pSDMA58 (contains hygromycin B resistance cassette), which is designed for targeted integration into a genomic “Safe Haven” location [25]. The *BTA1* locus was PCR-amplified from H99 WT genomic DNA using the primer pairs, BTA1-NotI-s and BTA1-XmaI-a. The PCR product was ligated into the XmaI and NotI sites of pSDMA58. The resulting pSDMA58_BTA1 plasmid was linearized with AscI and was introduced into *bta1Δ* by biolistic transformation. Hyg^r^ colonies were screened by multiplex PCR as described in [25] (**S2 Fig**). All primer sequences are listed in **S1 Table**.

### Spot dilution assay

*C*. *neoformans* strains were grown overnight at 30°C in YPD broth. Cells were pelleted by centrifugation, washed, and resuspended at a concentration of 10^6^ cells per 5 μl. Serial 10-fold dilutions were prepared and 5 μl of each suspension was spotted onto the various agar media. Macroscopic growth was recorded after 2–3 days of growth at 30°C.

### Lipid analysis by TLC

The *C*. *neoformans* WT and mutant strains, and the *C*. *albicans* WT strain (SC5314), were grown overnight in YPD broth. Cells were washed twice with water and resuspended at OD_600_ of 1 in either phosphate-replete (MM Pi+) or phosphate-deficient (MM Pi-) medium. Cultures were grown at 30°C overnight (18–24 hours). The OD_600_ of the cells was measured and the culture volume required to process the same amount of cells for each sample was calculated (OD_600_ 50–100). The cells were pelleted by centrifugation and snap-frozen in liquid nitrogen.

*Kluyveromyces lactis*, which was used as a control for DGTS production, was grown overnight on a YPD agar plate. Cells were scraped from the plate, washed twice with water and resuspended in either phosphate-free YNB medium (MP Biomedicals) supplemented with 0.5% glucose, or the same medium containing 29.4 mM KH_2_PO_4_. Cells were incubated with shaking at 30°C for 3 days, with the phosphate-containing cultures re-supplemented with fresh KH_2_PO_4_ every 24 hours at a concentration of 29.4 mM. Cells were collected by centrifugation and snap-frozen in liquid nitrogen.

The lipid extraction was performed in 2 ml O-ringed tubes. Water was added to each cell pellet to make a final volume of 300 μl and 1.2 ml of 100% ethanol was added. The cells in water:ethanol (1:4) mixture were then boiled for 45 minutes and centrifuged at 14,000 rpm for 1 minute to remove debris. The supernatant was transferred to a 15 ml tube and mixed with 4 ml chloroform, 4 ml methanol and 3.3ml of 0.2M KCl. Phase separation was achieved by centrifugation at 4,000 g for 10 minutes. The lower phase was washed twice with 7.6 ml of PBS:methanol (9:10) saturated with chloroform. After the final wash, the lower phase containing the lipid was dried under nitrogen gas. The dried lipids were dissolved in chloroform:methanol (2:1). Insoluble material was pelleted by centrifugation and the supernatant transferred to fresh tubes and used for one-dimensional thin layer chromatography (TLC) on silica plates. The solvent system used for lipid separation was chloroform:methanol:water (65:25:4). Lipids were visualized with I_2_ vapor and photographed immediately. Lipid standards were obtained from Sigma (PC, 1-Oleoyl-2-palmitoyl-*sn*-glycero-3-phosphocholine, Cat P-4142 and PE, L-α-Phosphatidylethanolamine, dioleoyl, Cat P-5078) and Avanti Polar Lipids (DGTS, 1,2-dipalmitoyl-sn-glycero-3-O-4'-(N,N,N-trimethyl)-homoserine, Cat. 857464).

### Lipid analysis using LC-MS

Lipids were extracted from fungal strains each adjusted to the same OD_600_ as described for the TLC analysis. Identification and comparative analysis of phospho- and betaine lipids was performed by LC-MS. Dried down lipid extracts were re-suspended in BuOH:MeOH (1:1 (v/v)) with 10 mM ammonium formate and separated by injecting 5 μL aliquots onto a 50 mm × 2.1 mm × 2.7 μm Ascentis Express RP Amide column (Supelco, Sigma, St Louis, USA) at 35°C using an Agilent LC 1200 (Mulgrave, Australia). The targeted lipid species were eluted at 0.2 mLmin^-1^ over a 5 min gradient of water/methanol/tetrahydrofuran (50:20:30, v/v/v) to water/methanol/tetrahydrofuran (5:20:75, v/v/v), with the final buffer held for 3 min. Lipids were analysed by electrospray ionisation-mass spectrometry (ESI-MS) using an Agilent Triple Quad 6410 (Mulgrave, Australia).

Lipid species from each lipid class (PC, PE, PS, PG, DGCC, DGTS and DGTA) were identified as previously described [26] using precursor ion scanning from 100–1000 m/z, in positive ion mode, phosphatidylcholines (PC, precursors of m/*z* 184.1), phosphatidylglycerols (PG, m/*z* 189), diacylglyceryl-N,N,N-trimethylhomoserine and diacylgyceryl hydroxymethyl-N,N,N-trimethyl-beta-alanine (DGTS and DGTA, precursors of m/*z* 236) and diacylglyceryl carboxyhydroxymethylcholine (DGCC, precursors of m/*z* 104) and in negative ion mode phosphatidylinositols (PI, m/*z* 241). Neutral loss scanning was used to identify phosphatidylethanolamines (PE, in positive ion mode, neutral loss of m/*z* 141) and phosphatidylserines (PS, negative ion mode, m/*z* 87). Although both DGTS and DGTA have the same multiple reaction monitoring (MRM) transitions due to their structural similarities, DGTS eluted later than DGTA on a chromatogram (see **S3 Fig**).

The levels of the identified lipid species were compared using MRM with a 20 ms dwell time for the simultaneous measurements of ~20 to 50 compounds and the chromatographic peak width of 30 sec to 45 sec, a minimum data points collected across the peak was 12 to 16. Optimised parameters for capillary, fragmentor, and collision voltages were 4000 V, 140–380, and 15–60 V, respectively. In all cases, the collision gas was nitrogen at 7 Lmin^-1^. Detected lipid species were annotated as follows; lipid class (sum of carbon atoms in the two fatty acid chains:sum of double bonds in the fatty acid chains). The LC/MS ESI-MRM data was processed using Agilent MassHunter quantitative software (version 6) (Mulgrave, Australia). Statistical analysis was performed using one way analysis of variance (ANOVA) where a p-value of <0.0001 was obtained and considered extremely significant. A pair-wise Tukey-Kramer multiple comparisons test was then performed.

### Virulence study

This study was approved and performed in accordance with the recommendations and guidelines of the Western Sydney Local Health District Animal Ethics Committee, Department of Animal Care, Westmead Hospital (protocol number 4254). Female C57BL/6 mice (20 to 22 g) were obtained from the Animal Resource Centre, Floreat Park, Western Australia, Australia. *C*. *neoformans* strains were grown overnight in YPD broth, washed twice with phosphate-buffered saline (PBS), and resuspended in PBS. Prior to infection, mice were transiently anesthetized by inhalation of 3% isoflurane, then 5x10^5^ cells in 20 μl of PBS were delivered into the nares using a pipette. For the survival analysis, 10 mice in each group were monitored daily and euthanized by CO_2_ asphyxiation when they had lost in excess of 20% of their pre-infection weight or if showing debilitating symptoms of infection. Differences in survival were determined using a log rank test. For organ burden analysis at the time of death, lungs and brain were then removed, weighed, and mechanically disrupted in 2 ml sterile PBS using a BeadBug (Benchmark Scientific). Serial dilutions of blood and the organ samples were plated onto SDA agar plates and incubated at 30°C for 2 days. Colony counts were performed and adjusted to reflect the total number of CFU per gram of tissue or ml of blood.

## Results and discussion

### Phosphate availability impacts the lipid profiles in wild-type *C*. *neoformans* and *Candida albicans*

**Fig 1:**
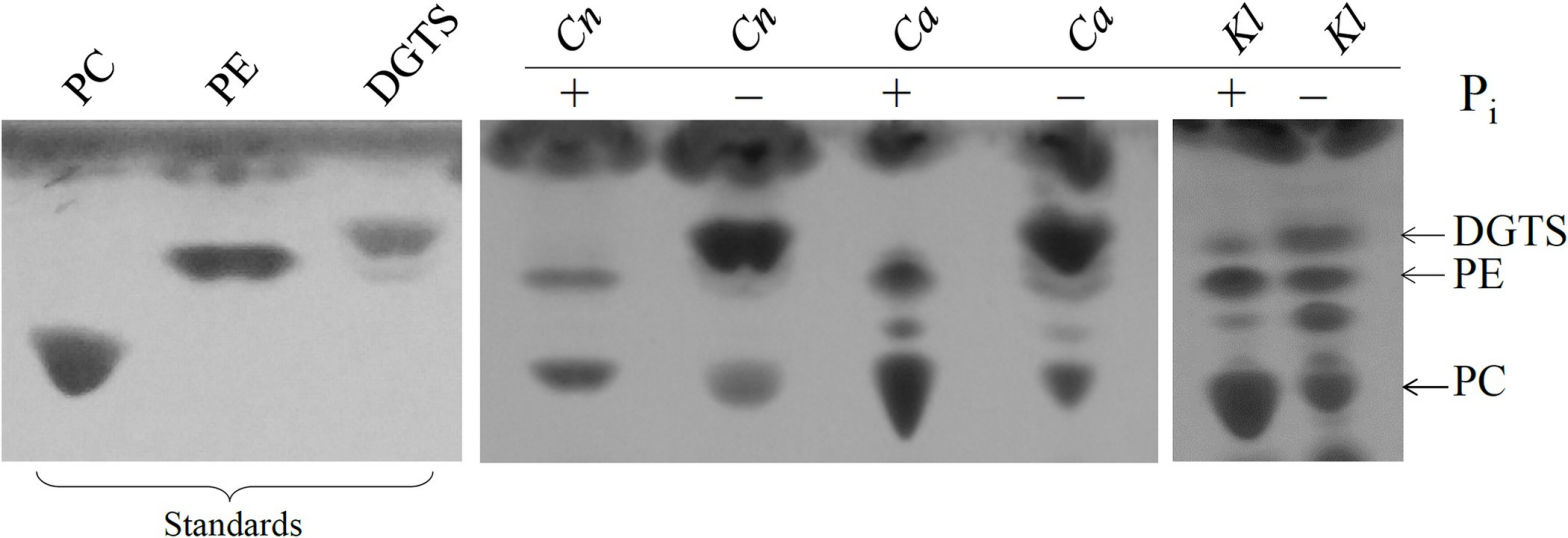
Impact of phosphate availability on the phospho- and betaine lipid profile in WT *C*. *neoformans (Cn)*, *C*. *albicans (Ca)* and *K*. *lactis (Kl)* as assessed by TLC. **(**Pi+): grown in the presence of phosphate; (Pi-) grown in the absence of phosphate. Lipids extracted from fungal cells were resolved by TLC and identified using commercially available standards. PE: phosphatidylethanolamine, PC: phosphatidylcholine, DGTS: diacylglyceryl-N,N,N-trimethylhomoserine.

To determine whether the lipid composition in *C*. *neoformans* changes in response to phosphate deprivation, WT was incubated in minimal medium in the presence and absence of phosphate and total lipids were isolated and analyzed by TLC **(Fig 1)**. We observed marked production of the phosphorus-free betaine lipid, DGTS, by phosphate-starved cells, and decrease in the amount of PC and PE. A similar pattern of lipid substitution was observed in *C*. *albicans* and *Kluyveromyces lactis* (**Fig 1**). We note however that *C*. *neoformans* and *C*. *albicans* produce more DGTS than *K*. *lactis* during phosphate starvation, suggesting that phospholipid replacement by betaine lipids is more efficient in these pathogens than in *K*. *lactis*. However, the lower rate of PC to DGTS conversion in *K*. *lactis* could be partially attributed to the different growth conditions, with phosphate-free YNB medium used instead of phosphate-free minimal medium and a longer incubation time of 3 days (see methods).

### The PHO pathway is required for PC substitution by DGTS in *C*. *neoformans*

**Fig 2:**
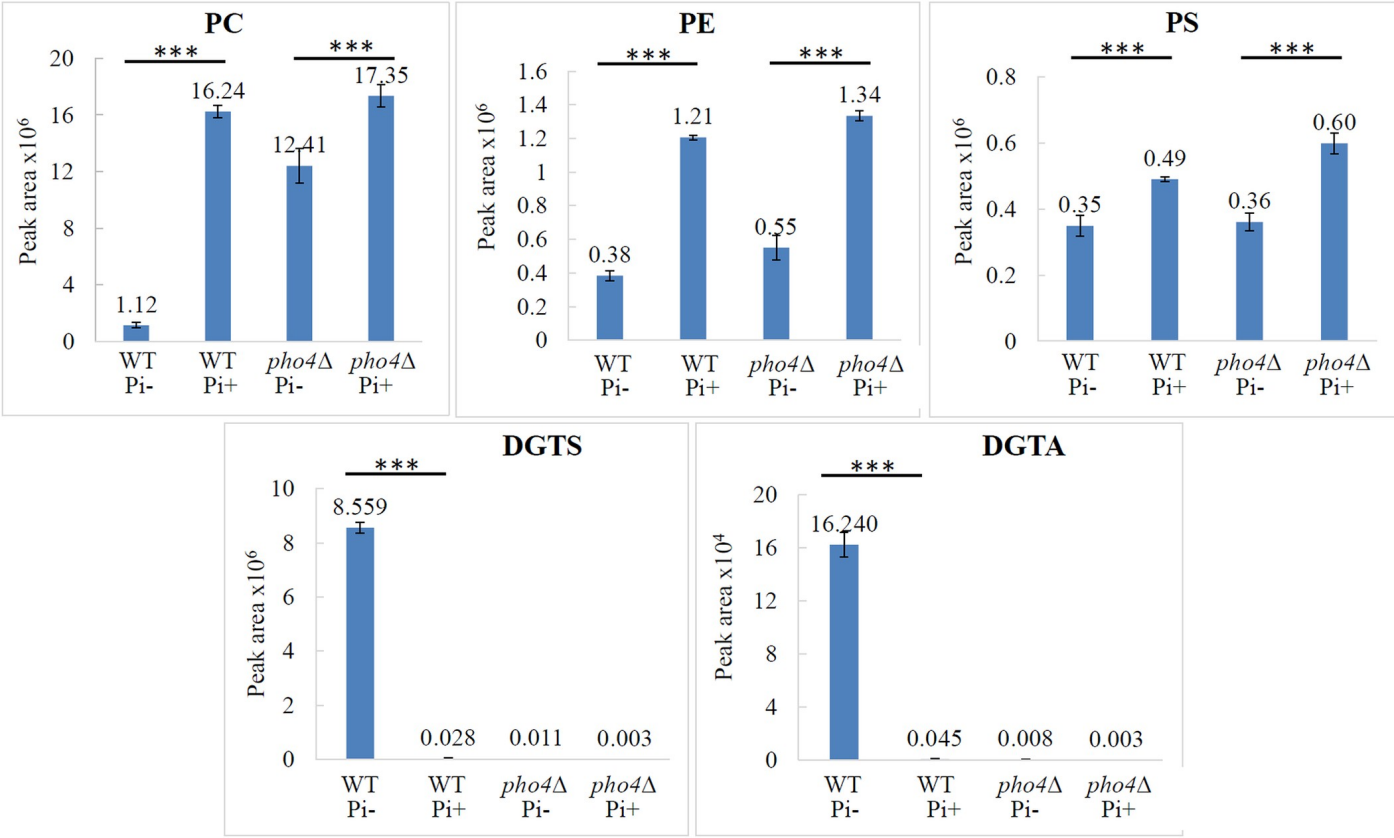
Effect of the PHO pathway on lipid profiles in *C*. *neoformans* as assessed by LC-MS. Cells were grown and their lipids extracted as in **Fig 1**. Graphs represent lipid profile comparisons of area under the curve and are the average of two biological replicates, each performed in technical duplicate. Error bars represent standard deviation. PE: phosphatidylethanolamine, PC: phosphatidylcholine, PS: phosphatidylserine, DGTS: diacylglyceryl-N,N,N-trimethylhomoserine, DGTA: diacylgyceryl hydroxymethyl-N,N,N-trimethyl-beta-alanine. ***p<0.001: calculated using one way ANOVA followed by a pair-wise Tukey-Kramer multiple comparisons test.

The lipid profiles in WT and the *pho4Δ* mutant were compared by LC-MS (**Fig 2)**. Due to fatty acid variation, LC-MS detects a range of masses for each lipid species **(S2 and S3 Tables).** All fatty acid variants within each lipid species were therefore included in the final quantification in **Fig 2.** Of the 3 major phospholipids produced in WT and the *pho4Δ* mutant when phosphate is present, PC is the most abundant, followed by PE and PS. This is consistent with the proportions observed by Singh et al [27]. In the absence of phosphate, the levels of PC, PE and PS in WT declined to 7%, 32% and 71% respectively, as compared to their levels in the presence of phosphate. Similar to the TLC results obtained in **Fig 1**, the significant decline in PC in WT during phosphate deprivation correlated with an approximately 300-fold increase in DGTS **(Fig 2)**. In addition to DGTS, another betaine lipid was present in phosphate-starved WT, with a mass consistent with that of DGTA. PE and PS levels declined in the phosphate-starved *pho4Δ* mutant to a similar level as that observed in WT. However, in contrast to WT, where the decline in PC was significant, PC levels only declined to 71% in phosphate-starved *pho4Δ*. Also in contrast to WT, the *pho4Δ* mutant produced very little DGTS or DGTA, irrespective of the cellular phosphate status. Taken together, the LC-MS data demonstrate that during phosphate starvation, WT *C*. *neoformans* conserves intracellular phosphate by producing the phosphorus-free betaine lipids, DGTS and DGTA instead of PC, and that this lipid interconversion process is Pho4-dependent. Furthermore, blocking PHO signaling during phosphate starvation has little effect on PE and PS levels relative to WT.

### PHO pathway-dependent *BTA1* is required for PC substitution by DGTS in *C*. *neoformans*

Data in **Fig 2** demonstrates that substitution of PC with phosphorus-free DGTS/DGTA is regulated by the transcription factor Pho4. We therefore investigated whether the Pho4-dependent gene *BTA1* (CNAG_02353), which is up-regulated >100-fold in WT *C*. *neoformans* during phosphate starvation [4,5], encodes the enzyme responsible for DGTS biosynthesis in *C*. *neoformans*. The putative cryptococcal Bta1 enzyme belongs to the S-adenosylmethionine-dependent methyltransferase superfamily and shares 36.6%, 34.5% and 36.4% identity with Bta1 orthologs in *K*. *lactis*, *N*. *crassa* and *C*. *albicans*, respectively. A *BTA1* gene deletion mutant (*bta1Δ*) was created by targeted homologous recombination (**S1 Fig**). We also created the corresponding rescue strain (*bta1Δ*+*BTA1*) using the “Safe-Haven” targeted ectopic integration method developed by the Fraser laboratory [25] (**S2 Fig**).

**Fig 3:**
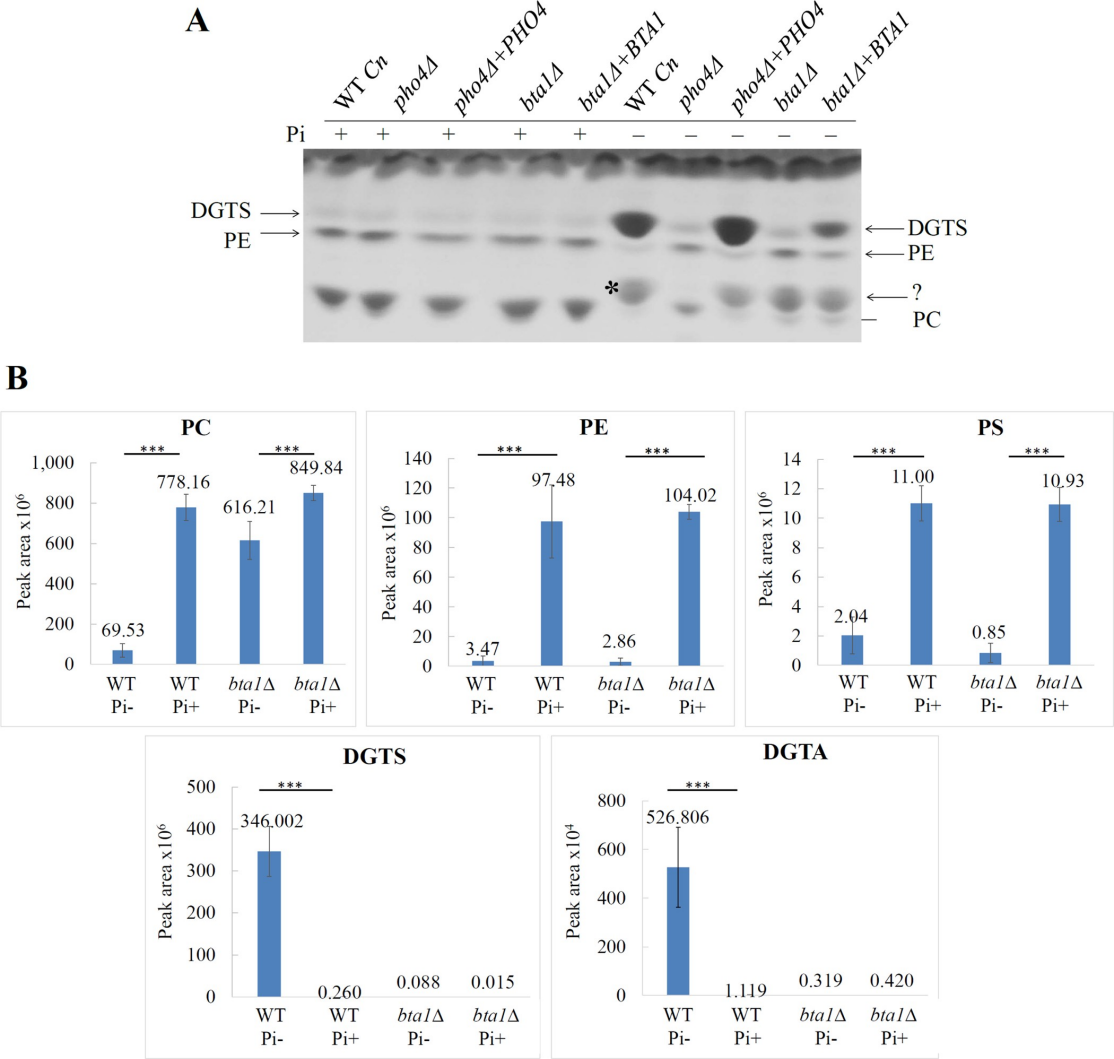
Impact of phosphate availability on the phospho- and betaine lipid profile in WT *C*. *neoformans (Cn)* and the *bta1Δ* mutant as assessed by TLC (A) and LC-MS (B). (Pi+): growth in the presence of phosphate; (Pi-): growth in the absence of phosphate. In (**A**), the *pho4Δ* and *bta1Δ* rescue strains, *pho4Δ*+*PHO4* and *bta1Δ*+*BTA1*, were included in the analysis as controls. Lipids isolated from each fungal strain were identified using commercially available standards. * indicates the position of an unidentified lipid produced in the absence of phosphate. In the LC-MS analysis in (**B**), the graphs represent lipid profile comparisons of area under the curve and are the average of two biological replicates, each performed in technical duplicate. Error bars represent standard deviation. PE: phosphatidylethanolamine, PC: phosphatidylcholine, PS: phosphatidylserine, DGTS: diacylglyceryl-N,N,N-trimethylhomoserine, DGTA: diacylgyceryl hydroxymethyl-N,N,N-trimethyl-beta-alanine. ***p<0.001: calculated using one way ANOVA followed by a pair-wise Tukey-Kramer multiple comparisons test.

WT, *bta1Δ* and the *bta1Δ*+*BTA1* strain were grown in minimal medium in the presence and absence of phosphate and lipids were extracted and resolved using TLC and LC-MS, as described for **Figs 1** and **2**. For TLC analysis **(Fig 3A)**, *pho4Δ* and its reconstituted strain (*pho4Δ+PHO4*) were also included for comparison. Similar to the LC-MS data **(Fig 2)**, the levels of DGTS in the phosphate-deprived *pho4Δ* mutant were low (**Fig 3A**). As expected, DGTS production was restored in *pho4Δ+PHO4* (**Fig 3A**). Similar to *pho4Δ*, DGTS content in *bta1Δ* did not increase in the absence of phosphate. DGTS production was partially restored in the *bta1Δ*+*BTA1* strain and this coincided with lower *BTA1* expression from its ectopic location, as indicated by qPCR (**S4 Fig**). Except for *pho4Δ*, an unidentified lipid was detected in all strains in the absence of phosphate (see asterisk). This lipid was not able to be revolved in **Fig 1**.

The lipid species in WT and *bta1Δ* were also quantified by LC-MS (**Fig 3B**). Similar to *pho4Δ* in **Fig 2**, production of DGTS was abrogated in the *bta1Δ* mutant, consistent with Pho4-dependent *BTA1* gene encoding a DGTS synthase. Production of DGTA was also compromised, suggesting that DGTS is a precursor of DGTA. Also similar to *pho4Δ* in **Fig 2**, phosphate deprivation led to a minor reduction in the level of PC in the *bta1Δ* mutant as compared to a large reduction in WT, while PE and PS levels declined significantly in both WT and *bta1Δ* (**Fig 3B**). We note the variability in PE and PS in WT cells in the presence and absence of phosphate in **Figs 2** and **3B**. However, despite this variability, the decline in PE and PS in WT in the absence of phosphate was similar to that observed in the *pho4Δ* and *bta1Δ* mutant strains. This is in contrast to PC where the decline in the WT in the absence of phosphate was always greater than the decline observed in the mutant strains in both experiments.

Taken together, the results in **Fig 3** demonstrate that DGTS production and PC breakdown inversely correlate and that *BTA1* encodes a functional DGTS synthase. They also show a positive correlation between DGTS and DGTA synthesis, consistent with DGTS being the precursor of DGTA. Substitution of PC with DGTS/DGTA is therefore both Pho4 and Bta1-dependent.

### Investigating whether the DAG backbone of PC is incorporated into DGTS and DGTA

The results in **Fig 3** suggest that PC is a precursor of DGTS. In algae and bacteria, Bta1 is an S-adenosylmethionine-dependent methyltransferase that uses DAG to synthesize DGTS [20,21]. As the DAG backbone is common to both phospho- and betaine lipids, we compared the fatty acid profiles of the phospholipids (PC, PE) and betaine lipids (DGTS, DGTA) obtained by LC-MS in **Figs 2** and **3** above. PE was included in the analysis because methylation of PE to produce PC (Greenberg pathway) is the predominant pathway by which PC is synthesised in yeast [28]. We reasoned that if the DAG backbone of PC is channeled into DGTS synthesis following PC hydrolysis, PC and DGTS would have similar FA profiles. The DAG backbone of each phospholipid species contains 2 fatty acids, which are not always identical. For example, PC can be comprised of a combination of palmitic (C16:0) and oleic acid (C18:1). Thus each lipid species quantified in **Figs 2** and **3** represents a mixture of isoforms that differ only in their fatty acids. As lipid determination was performed using LC-MS with a targeted multiple reaction monitoring (MRM) method, the individual fatty acids were not resolved for each isoform. For example, PC containing a combination of palmitic (C16:0) and oleic acids (C18:1) was identified as PC 34:1 (number of carbon atoms:number of unsaturated bonds, respectively).

**Fig 4:**
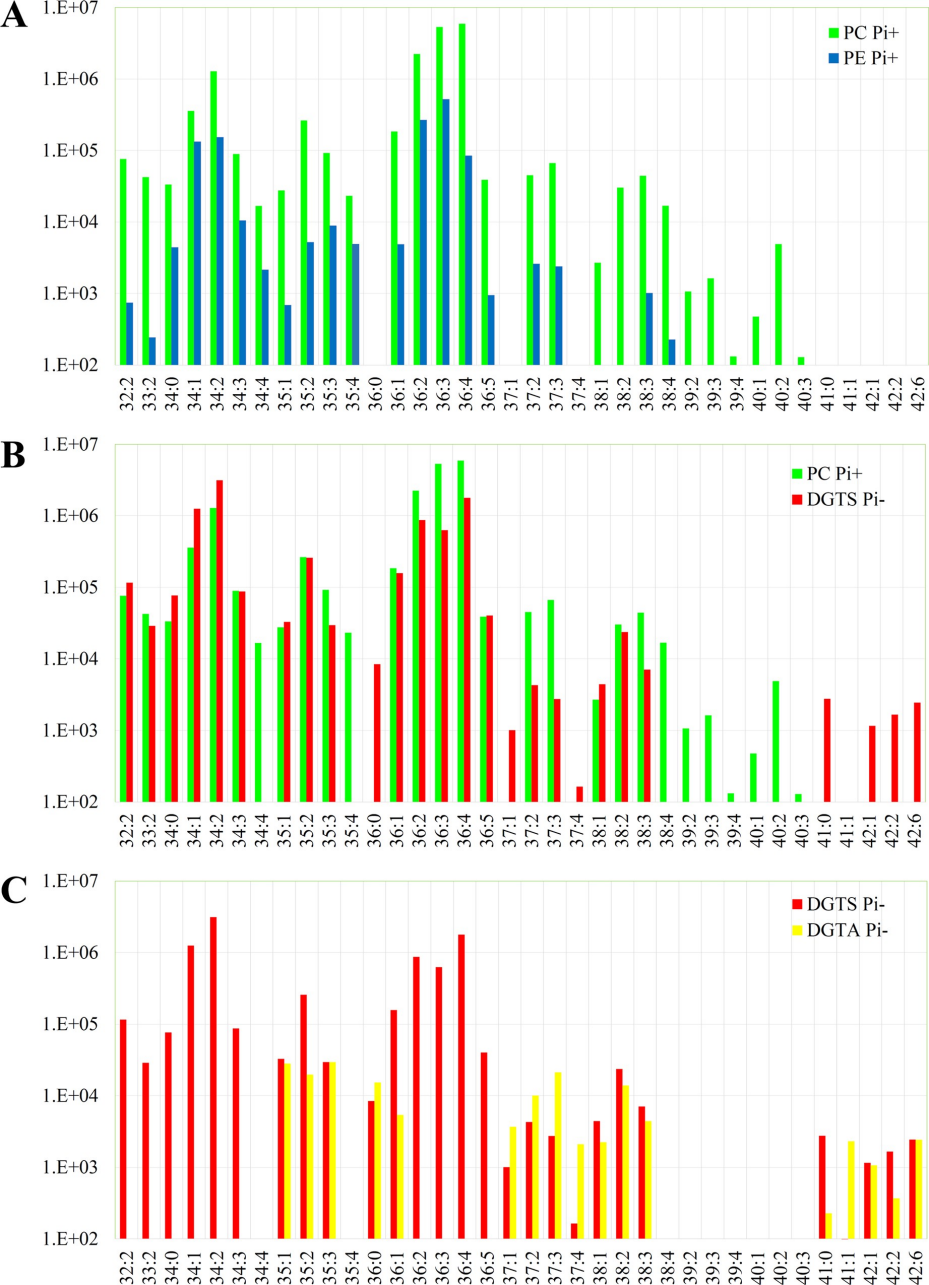
**Comparison of the fatty acid profiles in PE and PC (A), PC and DGTS (B) and DGTS and DGTA (C) as assessed by LC-MS.** Growth in the presence (Pi+) and absence (Pi-) of phosphate is indicated. PE: phosphatidylethanolamine, PC: phosphatidylcholine, DGTS: diacylglyceryl-N,N,N-trimethylhomoserine, DGTA: diacylgyceryl hydroxymethyl-N,N,N-trimethyl-beta-alanine. Numbers on the x-axis represent the combined number of carbon atoms in fatty acids of each lipid species: number of unsaturated bonds.

**Fig 5:**
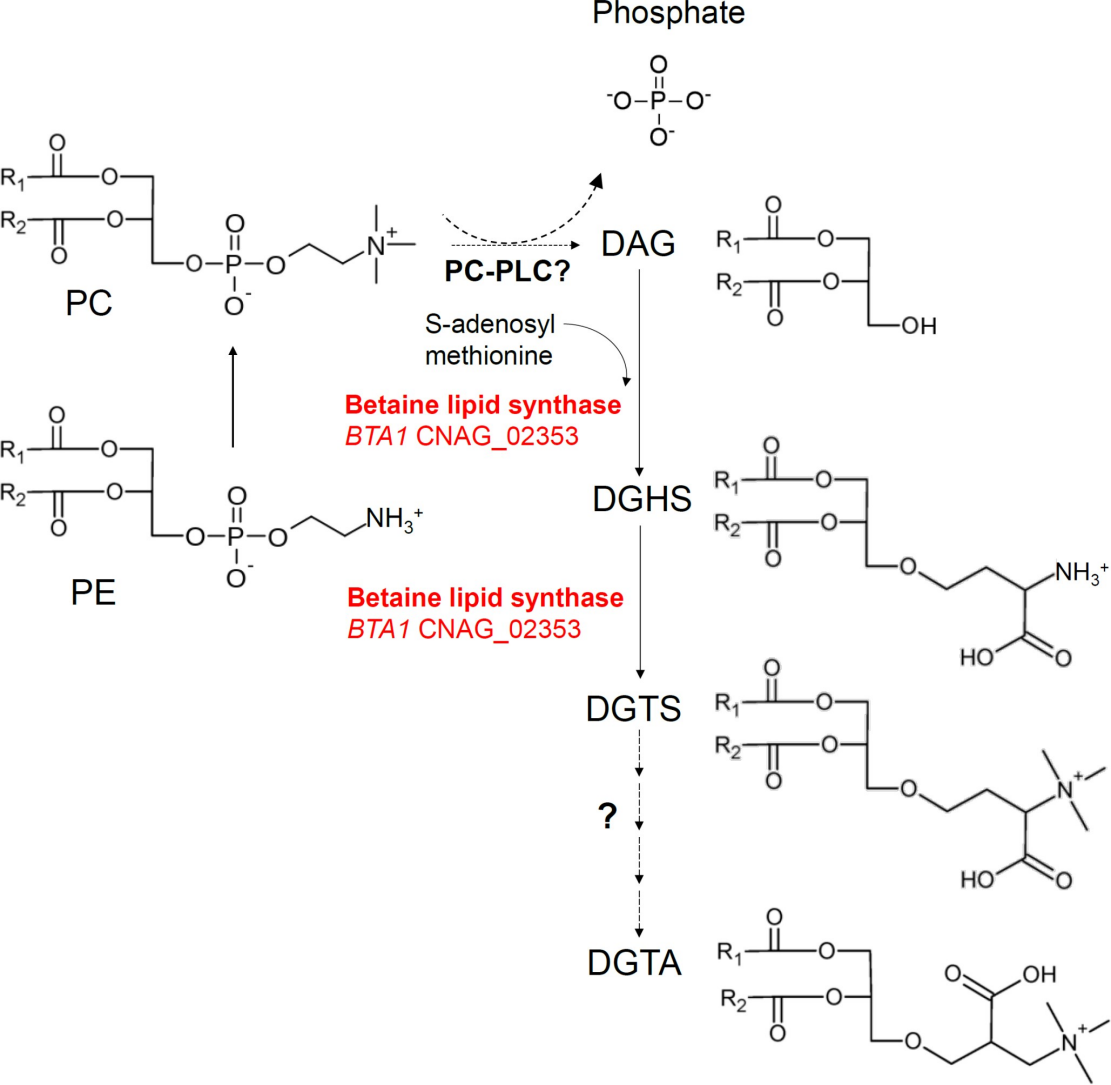
Model of phosphate recycling in *C*. *neoformans* via BTA1. Under phosphate-limiting conditions, phosphate may be recycled from PC via phospholipase C activity. We note however that an equivalent of the *F*. *graminearum* PC-PLC enzyme has not been identified in *C*. *neoformans* or *S*. *cerevisiae*. The DAG backbone of PC, which includes its fatty acids, is then incorporated into DGTS in a 2-step process via the betaine lipid synthase BTA1.

A comparison of the FA profiles of PC (Pi+) and PE (Pi+) showed that they correlated significantly (Spearman’s correlation coefficient r = 0.75) suggesting that, as has been reported in *S*. *cerevisiae*, most of the PC in *C*. *neoformans* is derived from PE via the methylation of PE (**Fig 4A**). Comparison of the FA profiles of PC (Pi+) and DGTS (Pi-) also revealed a positive correlation (r = 0.56). However, FA combinations C(38:4), C39 and C40, which are present in PC and PE, were absent in DGTS, and the opposite was observed for C41 and C42 (**Fig 4B**). We also compared the FA profiles of DGTS and DGTA. However, no positive correlation was observed (r = ^-^0.17), suggesting that the conversion of DGTS to DGTA involves FA remodeling **(Fig 4C)**. Predominantly, each DGTS species incorporated fatty acids with the combined number of carbons being even, while the combined number of carbons for DGTA was mostly odd. A model of phosphate recycling in *C*. *neoformans* via BTA1 is shown in **Fig 5.**

### Bta1 contributes to cryptococcal virulence in a mouse model

**Fig 6:**
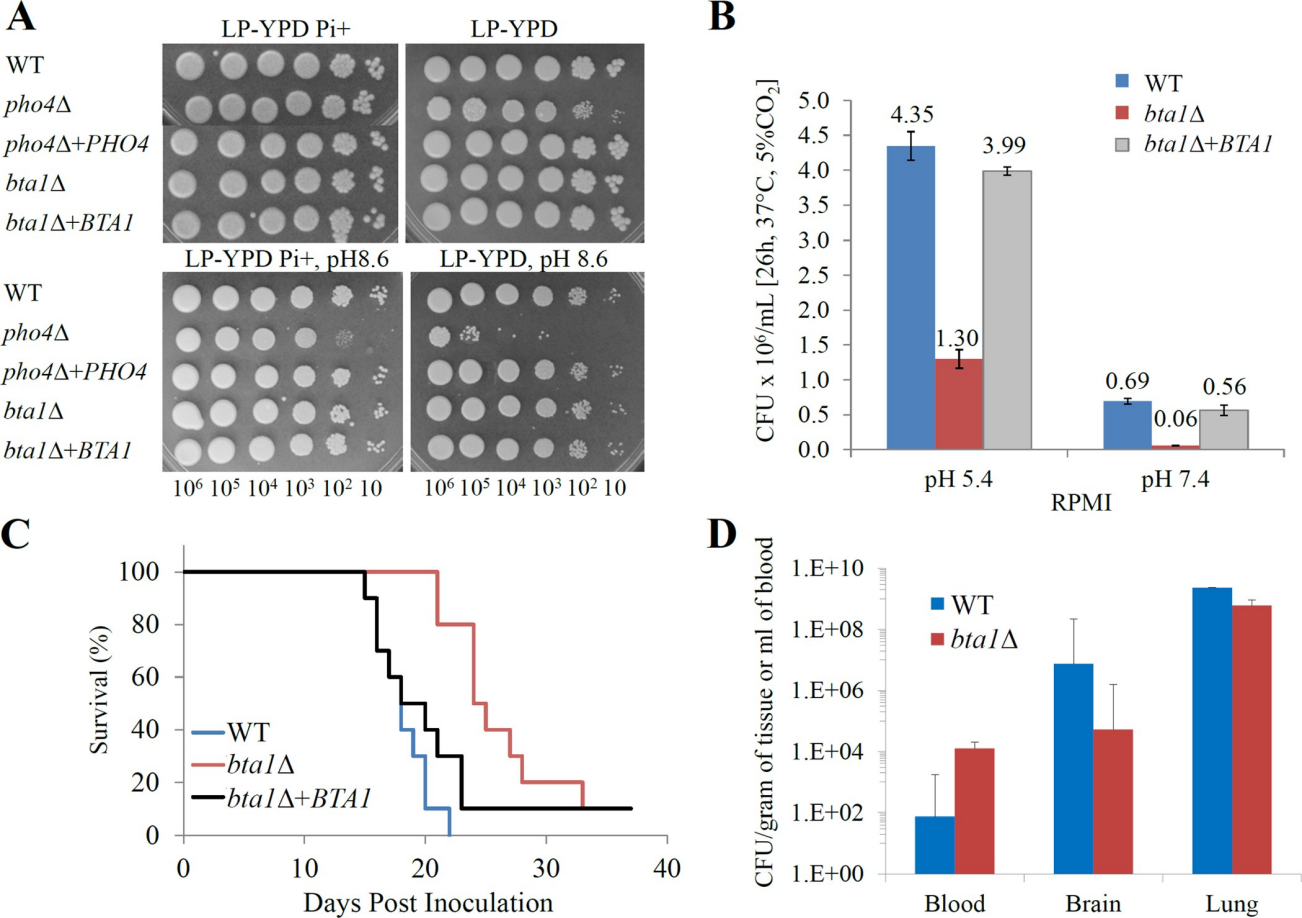
Bta1 is required for cryptococcal growth in mammalian cell culture medium and for virulence in a murine infection model. (A) Drop dilution assay: WT, *bta1Δ*, *pho4Δ* and the respective reconstituted strains were spotted onto low phosphate YPD (LP-YPD) with or without phosphate supplementation (29.4 mM KH_2_PO_4_). In one test, the pH was buffered at pH 8.6 as indicated. The plates were incubated for 2 days at 30°C (B) Quantitative culture in RPMI medium: WT, *bta1Δ* and *bta1Δ +BTA1* were grown in RPMI broth at 37°C in a 5% CO_2_ atmosphere for 26 hours. The pH was buffered at either 5.4 or 7.4 as indicated. Aliquots of the media were then plated onto SAB agar for CFU determination. (C) Survival study in a murine inhalation model: C57BL/6 mice were infected intranasally with 500,000 CFU of WT, *bta1Δ* or *bta1Δ +BTA1* (10 mice per group). *bta1Δ*-infected mice survived longer than WT- and *bta1Δ*+*BTA1*-infected mice (*P<*0.0001 and P = 0.03 respectively). The difference in survival between WT- and *bta1Δ +BTA1-*infected mice was not statistically significant (P = 0.1828). (D) Organ burden analysis from (C) at the time of illness: mean CFU from the lungs, brain (per gram of tissue) and blood (per ml) of 4–7 mice in each group that had succumbed to infection. Error bars indicate standard deviation.

We previously established that, relative to WT, the cryptococcal *pho4Δ* mutant grows more slowly in the absence of phosphate and that phosphate deprivation predisposes the mutant to hyper-susceptibility to cellular stresses including cell wall perturbing agents and antifungal drugs [4]. Notably, we found that the *pho4Δ* mutant is sensitive to alkaline pH regardless of phosphate availability [4]. To determine whether any of these phenotypes result from the absence of Bta1, we compared growth of WT and *bta1Δ* under the same stress conditions, in the presence and absence of phosphate. However, our findings demonstrate that inability to produce DGTS in *bta1Δ* effects neither its growth in the absence of phosphate nor its ability to tolerate cell wall perturbing agents, host temperature, antifungal drugs and lactose as an alternative carbon source with or without phosphate deprivation **(S5 Fig)**. Furthermore, *bta1Δ* tolerated alkaline pH regardless of phosphate status (**Fig 6A**) and was not deficient in any of the well characterized virulence factors, i.e. capsule, melanin and phospholipase B production (not shown). Interestingly, growth of *bta1Δ* was compromised in cell culture media (RPMI), but unlike *pho4Δ*, it was only partially restored by lowering the pH of the medium to 5.4 (**Fig 6B**).

Given that the *bta1Δ* mutant has a growth defect in mammalian cell culture medium, we tested the virulence of *bta1Δ* in a murine inhalation model. **Fig 6C** shows that *bta1Δ*-infected mice survived 6 days longer than WT-infected animals (24 days *vs* 18 days, respectively, p<0.0001), and 4 days longer than the *bta1Δ*+*BTA1*-infected mice (mean survival 20 days, p = 0.03). However unlike *pho4Δ*, *bta1Δ* still disseminated to the brain: at the time when the animals succumbed to infection, no significant difference in CFU in the lung, blood or brain of WT- and *bta1Δ*-infected mice was observed **(Fig 6D)**.

In this study we demonstrate that activation of the cryptococcal PHO pathway conserves phosphate via replacement of PC with DGTS and DGTA. To our knowledge, this is the first report of DGTA production by *C*. *neoformans* and by fungi in general. DGTA may not have been detected previously in other fungi due to its low abundance, use of insensitive methods of detection and/or the fact that fungi are usually cultured in nutrient replete medium [18,19]. We also found that the decline in PC during phosphate starvation, but not the decline of PE and PS, is contingent on both a functional PHO pathway and PHO-pathway mediated induction of *BTA1*. Thus, PE and PS act as phosphate donors during phosphate deprivation and their ability to relinquish phosphate is independent of a functional PHO pathway and/or a functional DGTS synthase. Our observations support the proposition that PC is the precursor of DGTS. The inverse correlation between PC and DGTS, as orchestrated by the PHO pathway, also occurs in *Neurospora crassa* [17] and we now show that the ascomyceteous opportunistic fungal pathogen *C*. *albicans* also substitutes PC for DGTS in response to phosphate deprivation.

We also investigated mechanisms of conversion of PC to DGTS. The fact that the levels PC are strictly maintained in *C*. *neoformans* in the absence of a functional PHO pathway and/or a functional Bta1 enzyme, suggests that a tightly linked mechanism operates in *C*. *neoformans* to coordinate PC hydrolysis with DGTS synthesis. Given that PC is the most abundant phospholipid in fungal membranes the presence of this linked mechanism is most likely required to conserve fungal membrane integrity during phosphate deprivation. Our observation that PC and DGTS have a similar FA composition suggests that the DAG backbone of PC is utilised to synthesise DGTS, not the fatty acids released from it. Indeed, this was shown to be the case in the bacterium *Sinorhizobium meliloti* [10], the model plant *Arabidopsis thaliana* [29] and the plant fungal pathogen *F*. *graminearum* [22]. In these microorganisms, PC is hydrolysed by a phospholipase C to release phosphocholine and DAG. DAG is then reutilized to produce either DGTS in fungi or galactolipids in plants. However, the exact mechanism by which DAG is released from PC in *C*. *neoformans* remains to be investigated as no PC-PLC homolog has been identified in the cryptococcal genome. Interestingly, although PC-PLC activity is involved in cell cycle regulation in *S*. *cerevisiae*, the enzyme responsible for this activity has also not been identified [30]. It is also possible that PC conversion to DGTS is a redundant process in many fungi, with the generation of DAG involving numerous hydrolases e.g. phospholipase D (*PLD1* and *SPO14*) and glycerophosphodiesterases (*GDE1*, *GDE2* and *GDE3*), all of which would be expected to conserve the DAG backbone for incorporation into DGTS. Finally, it cannot be ruled out that a proportion of DGTS is derived from the DAG storage pool given that FA correlation between PC and DGTS was not 100%.

We established that in addition to DGTS, Bta1 is essential for production of DGTA. However, the FA profiles of DGTS and DGTA did not correlate, suggesting that the DAG backbone in DGTS is not retained during the conversion. In algae, DGTA is converted to DGTS in a multistep process involving decarboxylation and recarboxylation of the polar head group, followed by a simultaneous deacylation and reacylation of the glycerol moiety [16,31]. Extensive FA differences between DGTA and DGTA in *C*. *neoformans* support the hypothesis that a similar mechanism operates in this yeast.

Similar to *pho4Δ* [4], *bta1Δ* was hypovirulent in a mouse model. A comparison of the phenotypes of *pho4Δ* and *bta1Δ* showed that *bta1Δ* hypovirulence is most likely due to its delayed growth under physiological conditions (exclusive of alkaline pH). Despite this delayed growth, *bta1Δ* still disseminated to the brain. This was in contrast to *pho4Δ*, which showed significantly less dissemination to the brain [4]. DGTS synthase was also dispensable for growth in the absence of phosphate, suggesting that the phosphate liberated by the substitution of PC with DGTS does not contribute significantly to fungal growth during phosphate deprivation.

In contrast, the reduced virulence of the *bta1Δ* mutant in *F*. *graminearum* is directly associated with its inability to produce DGTS and sustain intercellular growth in the phosphorus-depleted maize apoplast [22]. We therefore considered two alternative explanations for the importance of Bta1 in cryptococcal virulence. Firstly, under physiological condition, Bta1 may have a function independent of its catalytic activity, for example by acting as a structural component or a scaffold for lipid processing. Alternatively, betaine lipids may affect membrane organisation and associated virulence factors through lipid-lipid and lipid-protein interactions. In support of this hypothesis, *C*. *neoformans* produces detectable basal levels of betaine lipid even when phosphate is high (29 mM) **(Figs 2 and 3)**. Thus, although the abundance of DGTS/DGTA in fungal cells may be relatively low during host infection, it may still impact virulence. Additionally, DGTS/DGTA may incorporate into fungal microvesicles and alter their immunogenic properties, especially since DGTS/DGTA are not produced by the mammalian host. Interestingly, betaine lipids, including DGTS, have been reported to inhibit NO production in RAW264.7 macrophages by down-regulating expression of nitric oxide synthase. Based on this observation, betaine lipids are considered as potential anti-inflammatory agents [32].

## Conclusions

In summary, we have identified a PHO pathway-regulated betaine lipid synthase in a fungal pathogen of medical significance, which is involved in phosphate recycling from PC and is absent in the model yeast *S*. *cerevisiae*. Bta1 allows substitution of PC with DGTS as a fail-safe mechanism to maintain both membrane integrity and intracellular phosphate levels and contributes to cryptococcal virulence in an animal model.

## Supporting information

S1 Table Primers used for creating *bta1Δ* and *bta1Δ*+*BTA1* strains, and for verification of mutants in genes encoding lipid remodeling enzymes. (PDF)

S2 Table LC-MS data pho4. (XLSX)

S3 Table LC-MS data bta1. (XLSX)

S1 Fig Screening of the transformants by PCR to identify *bta1Δ* mutants. Integration of the deletion construct was verified across 5’ and 3’ recombination junctions. The verification PCR at the 3’ end was performed using primers BTA1-3’-ots and Ttrp-s (expected size = 1165bp), while the 5’ verification PCR was performed using primers BTA1-5’-new1-s and ActP-a (expected size = 896bp). WT was included to confirm that the products were *bta1Δ*-specific. All primers sequences are listed in **S1 Table**.
(PDF)

S2 Fig Verification of the targeted integration of AscI-linearized pSDMA58_BTA1 into *bta1Δ* genome (“Safe Haven” site). Multiplex PCR was performed using primers within the pSDMA58_BTA1 (UQ1768 and UQ3348) and primers outside of pSDMA58_BTA1 (UQ2962 and UQ2963), therefore yielding 2 bands with different size: 1514bp from primer combination UQ2962 and UQ3348, and 1203bp from primer combination UQ1768 and UQ2963. pSDMA58_BTA1 integrated into the “Safe Haven” in transformants 1, 3, 4, and 5. *bta1Δ* was included as a negative control (expected size of the single band = 2177bp). Sequences of primers are listed in **S1 Table**.
(PDF)

S3 Fig **LC-MS chromatogram for (A) DGTS(36:4) (RT = 9.5 min) and (B) DGTA(36:4) (RT = 8.5 min).** DGTS elutes later than DGTA even though both have the same MRM profile.
(PDF)

S4 Fig Quantitation of *BTA1* expression levels using qPCR. Each strain was grown in minimal media in the presence and absence of phosphate (Pi) for 3 h. The cells were homogenized by bead beating in the presence of glass beads and TRIzol (Ambion) and RNA was extracted following the manufacturer’s instructions. cDNA was synthesized using Moloney murine leukemia virus reverse transcriptase (Promega). Specific transcripts were quantified by quantitative PCR (qPCR) using the SYBR green real-time PCR master mix (Life Technologies) on a Rotorgene 6000 qPCR machine (Corbett Research). Gene expression was normalized against the expression of actin (*ACT1*) as a housekeeping gene before final quantification using the 2^−*ΔΔCT*^ calculation method. The results are expressed as fold-change relative to WT H99 + Pi.
(PDF)

S5 Fig Spot dilution assay demonstrates that *bta1Δ* and WT *C*. *neoformans* are similarly tolerant to stresses. Plates were prepared using YNB without phosphate as a base. Pi+ plates were supplemented with 29.4 mM KH_2_PO_4_, and Pi- plates were supplemented with 29.4 mM KCl. Strains were tested for their ability to grow at 37°C, in the presence of Amphotericin B and cell wall perturbing agents (Congo Red, Calcofluor White and SDS). To test the ability of the *bta1Δ* to assimilate carbon sources other than glucose, the glucose was replaced with lactate.
(PDF)

## Abbreviations

TLC: thin layer chromatography
LC-MS: liquid chromatography mass spectrometry
PC: phosphatidylcholine
PS: phosphatidylserine
PE: phosphatidylethanolamine
DGTS: diacylglyceryl-N,N,N-trimethylhomoserine
DGTA: diacylgyceryl hydroxymethyl-N,N,N-trimethyl-beta-alanine
