# Supplementary material for: The PHO signaling pathway directs lipid remodeling in Cryptococcus neoformans via DGTS synthase to recycle phosphate during phosphate deficiency
Source: PLoS One. 2019 Feb 21;14(2):e0212651. doi: 10.1371/journal.pone.0212651 (PMC6383925; doi:10.1371/journal.pone.0212651)
Supplement: S1 Table — (PDF) [file pone.0212651.s001.pdf]

Table S1. Primers used for creating *bta1*Δ and *bta1*Δ+*BTA1* strains, and for verification of mutants in genes encoding lipid remodeling enzymes.

**Description**	**Name**	**Sequence**	**Name**	**Sequence**
5' flanking region for homologous recombination to delete *BTA1* gene	BTA1-5'new2-s	GACTATCGTTCTCACCACGGCTCA	BTA1-5'CDS-a	CTCCAGCTCACATCCTCGCAGGCATTAACGGGAAGCCTCACAA
3' flanking region for homologous recombination to delete *BTA1* gene	BTA1-3'-s	TCCTCAGGATCTTCATGGCTCC GGTGATGGAGTAGGAAATATAGGGTG	BTA1-3'-a	CTCTCGCTCTCGCTCTGGCTC
Amplification of neomycin resistance cassette	Neo-s	CTGCGAGGATGTGAGCTGGAG	Neo-a	GGAGCCATGAAGATCCTGAGG
Verification PCR amplifying external 3' of recombination region	BTA1-3'-ots	TGACGAGCTTCGGGATACTGTG	Ttrp-s	CTACAGACAACAATACCATCCTTCC
Verification PCR amplifying external 5' of recombination region	BTA1-5'new1-s	CACGTTCGACCATTCACAAGCA	ActP-a	TGTTGTTACCATCATCCTCTCCTC
Amplification of *BTA1* CDS for cloning into pSDMA58	BTA1-NotI-s	tgct GCGGCC GCCACGTTCGACCATTCACAA	BTA1-XmaI-a	agtt cccggg TGAGCAAAGTCGGTTGTCTAGCAT
Multiplex PCR for verification of targeted integration of *BTA1* to Safe Haven site	UQ1768	TCAGCAACGCCGTTGAATCCT	UQ2962	GGGTATGCCACAGATGCAGAT
	UQ2963	TTGGATCCTCAATTGTCTCCT	UQ3348	ACTGGTGAGTACTCAACCAAG
Verification of the various deletion mutants by RT-PCR	PAP2-s	GTGCGGTGATTGGGCTGTTG	PAP2-a	GGGACGAGAATGAGAATGGAGTGG
	GDE2-s	GGGGTATTGCCAGTGTCATTTCAGA	GDE2-a	TATTCCGTTTCTTCCTTGGCGAG
	PLD1-s	GTCTACTACCCTCGCAGCCAGC	PLD1-a	GGCATGCGGTTTCAAGTATTCCT
	NTE1-s	CACTCCCATTCAGCCTTTGCG	NTE1-a	CCTCCTCCTTCCACTTCTTCAACG
	GDE3-s	CGCCTTCTGATACCCTGCCTGAT	GDE3-a	TCCTTCGCCTGACTCCCTTGC
