# Supplementary material for: The PHO signaling pathway directs lipid remodeling in Cryptococcus neoformans via DGTS synthase to recycle phosphate during phosphate deficiency
Source: PLoS One. 2019 Feb 21;14(2):e0212651. doi: 10.1371/journal.pone.0212651 (PMC6383925; doi:10.1371/journal.pone.0212651)
Supplement: S1 Fig — Integration of the deletion construct was verified across 5’ and 3’ recombination junctions. The verification PCR at the 3’ end was performed using primers BTA1-3’-ots and Ttrp-s (expected size = 1165bp), while the 5’ verification PCR was performed using primers BTA1-5’-new1-s and ActP-a (expected size = 896bp). WT was included to confirm that the products were bta1Δ-specific. All primers sequences are listed in S1 Table. (PDF) [file pone.0212651.s004.pdf]

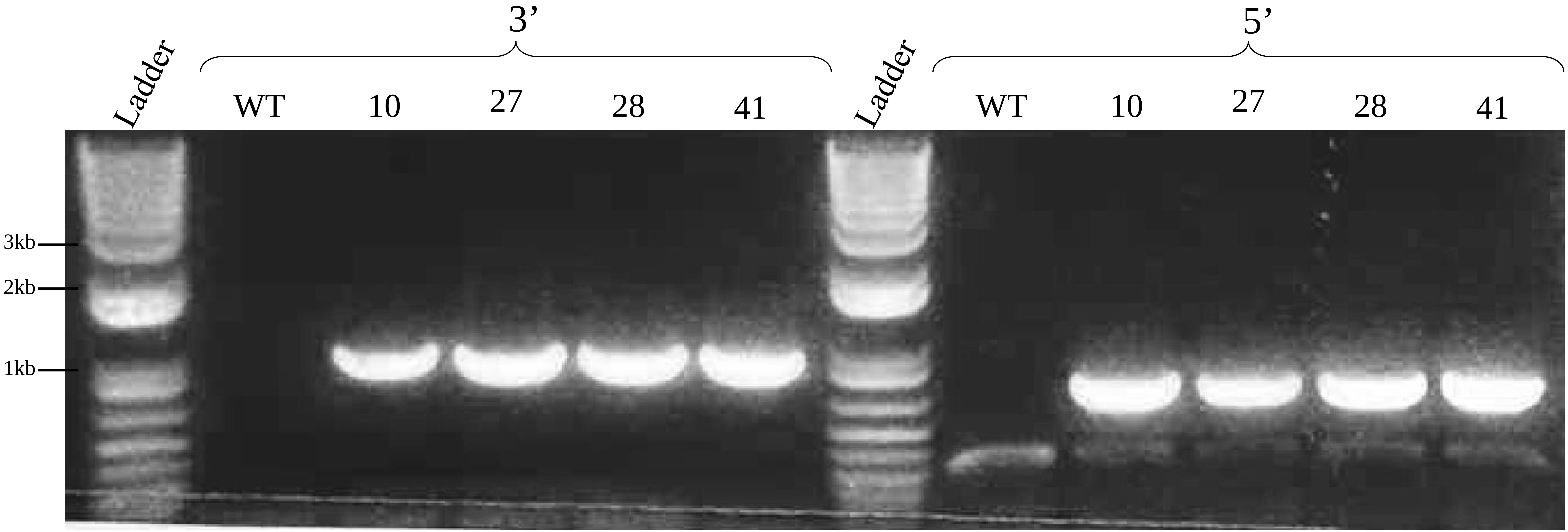

Figure S1. Screening of the transformants by PCR to identify *bta1*Δ mutants. Integration of the deletion construct was verified across 5’ and 3’ recombination junctions. The verification PCR at the 3’ end was performed using primers BTA1-3’-ots and Ttrp-s (expected size = 1165bp) , while the 5’ verification PCR was performed using primers BTA1-5’-new1-s and ActP-a (expected size = 896bp). WT was included to confirm that the products were *bta1*Δ-specific. All primers sequences are listed in **Table S1**.
