# Supplementary material for: The PHO signaling pathway directs lipid remodeling in Cryptococcus neoformans via DGTS synthase to recycle phosphate during phosphate deficiency
Source: PLoS One. 2019 Feb 21;14(2):e0212651. doi: 10.1371/journal.pone.0212651 (PMC6383925; doi:10.1371/journal.pone.0212651)
Supplement: S2 Fig — Multiplex PCR was performed using primers within the pSDMA58_BTA1 (UQ1768 and UQ3348) and primers outside of pSDMA58_BTA1 (UQ2962 and UQ2963), therefore yielding 2 bands with different size: 1514bp from primer combination UQ2962 and UQ3348, and 1203bp from primer combination UQ1768 and UQ2963. pSDMA58_BTA1 integrated into the “Safe Haven” in transformants 1, 3, 4, and 5. bta1Δ was included as a negative control (expected size of the single band = 2177bp). Sequences of primers are listed in S1 Table. (PDF) [file pone.0212651.s005.pdf]

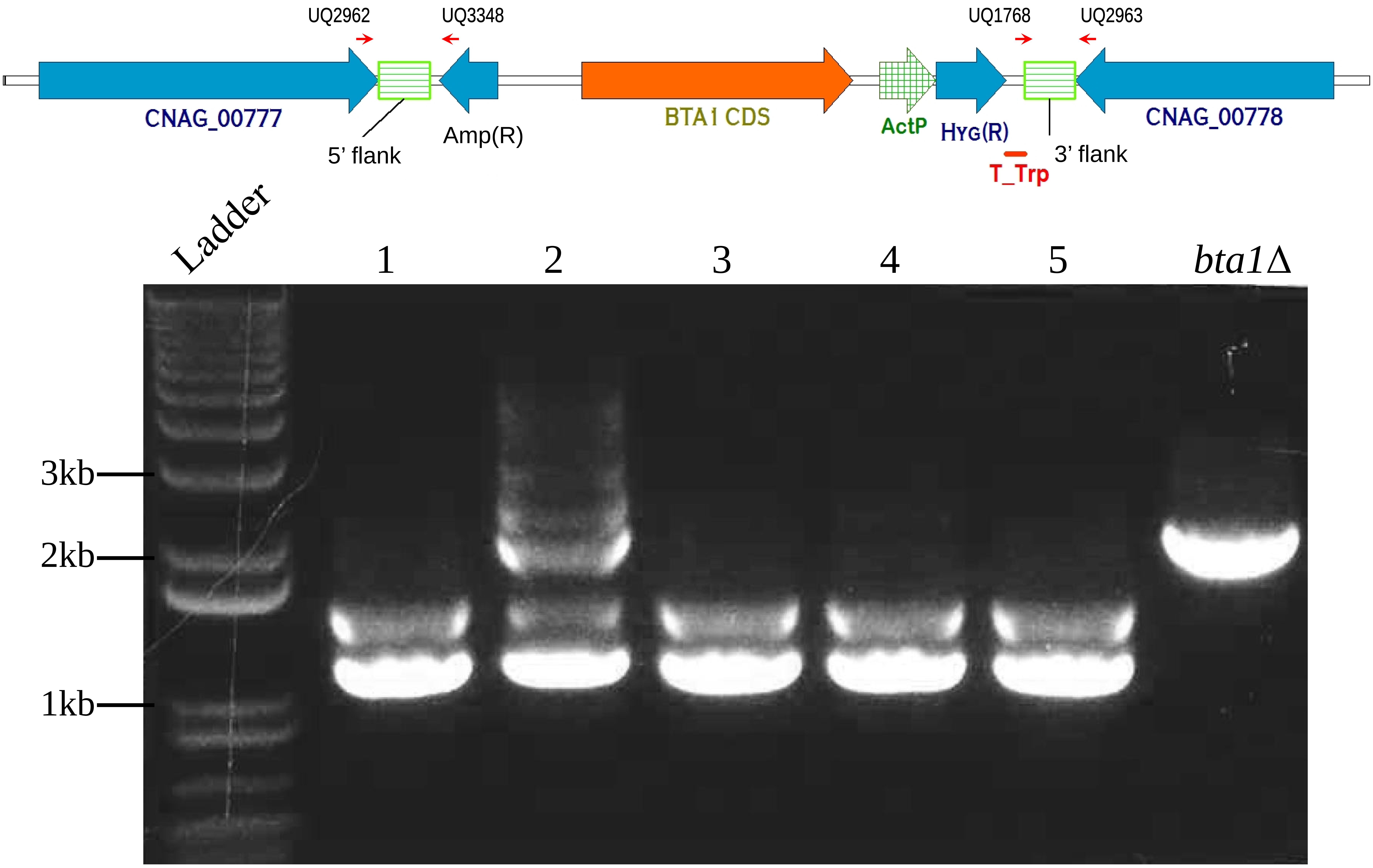

S2_Figure. **Verification of the targeted integration of AscI-linearized pSDMA58_BTA1 into *bta1*Δ genome ("Safe Haven" site)**. Multiplex PCR was performed using primers within the pSDMA58_BTA1 (UQ1768 and UQ3348) and primers outside of pSDMA58_BTA1 (UQ2962 and UQ2963), therefore yielding 2 bands with different size: 1514bp from primer combination UQ2962 and UQ3348, and 1203bp from primer combination UQ1768 and UQ2963. pSDMA58_BTA1 integrated into the "Safe Haven" in transformants 1, 3, 4, and 5. *bta1*Δ was included as a negative control (expected size of the single band = 2177bp). Sequences of primers are listed in **Table S1**.
