# Supplementary material for: The PHO signaling pathway directs lipid remodeling in Cryptococcus neoformans via DGTS synthase to recycle phosphate during phosphate deficiency
Source: PLoS One. 2019 Feb 21;14(2):e0212651. doi: 10.1371/journal.pone.0212651 (PMC6383925; doi:10.1371/journal.pone.0212651)
Supplement: S3 Fig — LC-MS chromatogram for (A) DGTS(36:4) (RT = 9.5 min) and (B) DGTA(36:4) (RT = 8.5 min). DGTS elutes later than DGTA even though both have the same MRM profile. (PDF) [file pone.0212651.s006.pdf]

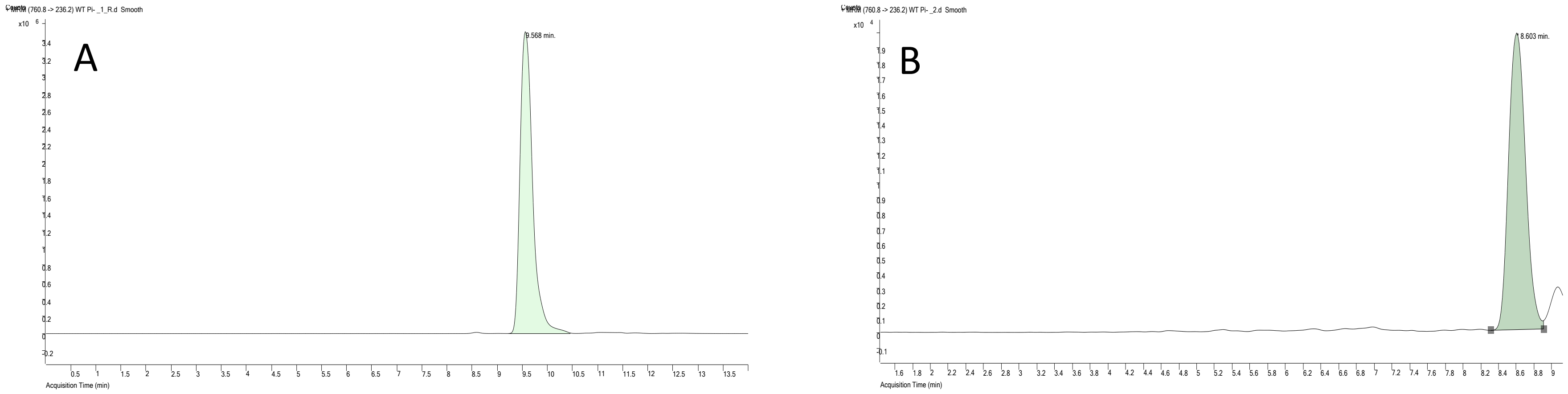

S3_Figure. **LC-MS chromatogram for (A) DGTS(36:4) (RT=9.5 min) and (B) DGTA(36:4) (RT=8.5 min)**. DGTS elutes later than DGTA even though both have the same MRM profile.
