# Supplementary material for: The PHO signaling pathway directs lipid remodeling in Cryptococcus neoformans via DGTS synthase to recycle phosphate during phosphate deficiency
Source: PLoS One. 2019 Feb 21;14(2):e0212651. doi: 10.1371/journal.pone.0212651 (PMC6383925; doi:10.1371/journal.pone.0212651)
Supplement: S4 Fig — Each strain was grown in minimal media in the presence and absence of phosphate (Pi) for 3 h. The cells were homogenized by bead beating in the presence of glass beads and TRIzol (Ambion) and RNA was extracted following the manufacturer’s instructions. cDNA was synthesized using Moloney murine leukemia virus reverse transcriptase (Promega). Specific transcripts were quantified by quantitative PCR (qPCR) using the SYBR green real-time PCR master mix (Life Technologies) on a Rotorgene 6000 qPCR machine (Corbett Research). Gene expression was normalized against the expression of actin (ACT1) as a housekeeping gene before final quantification using the 2−ΔΔCT calculation method. The results are expressed as fold-change relative to WT H99 + Pi. (PDF) [file pone.0212651.s007.pdf]

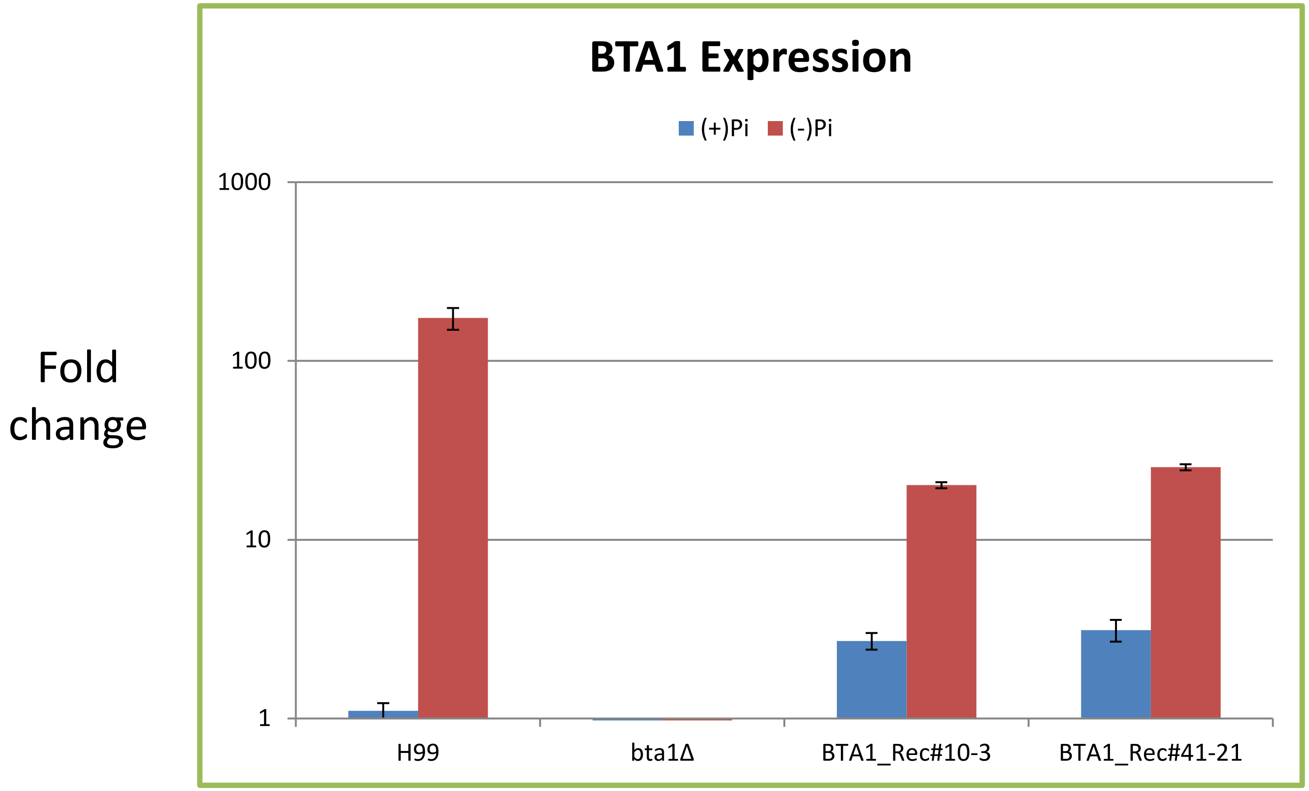

S4_Figure. Quantitation of *BTA1* expression levels using qPCR. Each strain was grown in minimal media in the presence and absence of phosphate (Pi) for 3 h. The cells were homogenized by bead beating in the presence of glass beads and TRIzol (Ambion) and RNA was extracted following the manufacturer's instructions. cDNA was synthesized using Moloney murine leukemia virus reverse transcriptase (Promega). Specific transcripts were quantified by quantitative PCR (qPCR) using the SYBR green real-time PCR master mix (Life Technologies) on a Rotorgene 6000 qPCR machine (Corbett Research). Gene expression was normalized against the expression of actin (*ACT1*) as a housekeeping gene before final quantification using the $2^{-\Delta\Delta CT}$ calculation method. The results are expressed as fold-change relative to WT H99 + Pi.
