# Supplementary material for: The PHO signaling pathway directs lipid remodeling in Cryptococcus neoformans via DGTS synthase to recycle phosphate during phosphate deficiency
Source: PLoS One. 2019 Feb 21;14(2):e0212651. doi: 10.1371/journal.pone.0212651 (PMC6383925; doi:10.1371/journal.pone.0212651)
Supplement: S5 Fig — Plates were prepared using YNB without phosphate as a base. Pi+ plates were supplemented with 29.4 mM KH2PO4, and Pi- plates were supplemented with 29.4 mM KCl. Strains were tested for their ability to grow at 37°C, in the presence of Amphotericin B and cell wall perturbing agents (Congo Red, Calcofluor White and SDS). To test the ability of the bta1Δ to assimilate carbon sources other than glucose, the glucose was replaced with lactate. (PDF) [file pone.0212651.s008.pdf]

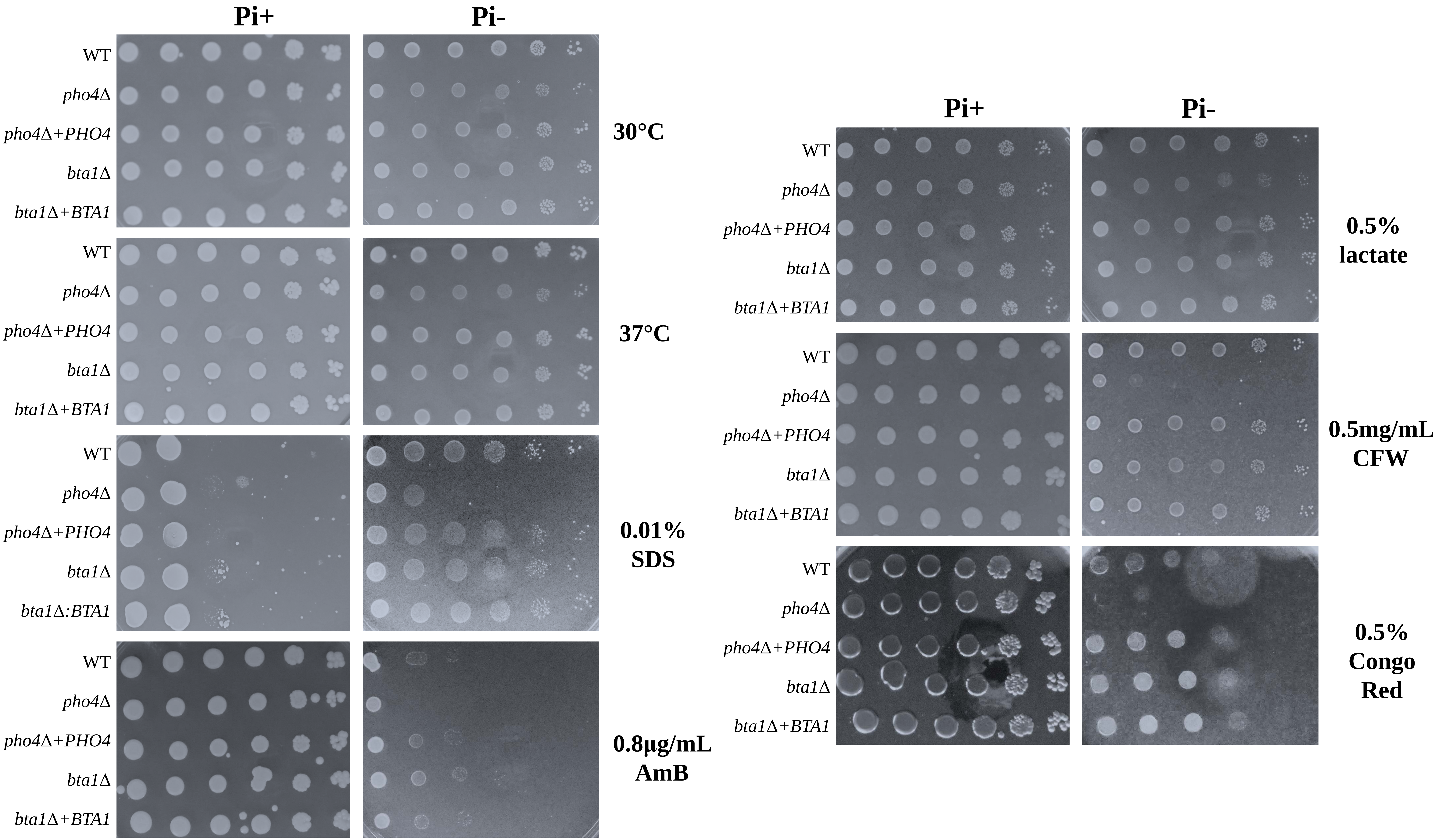

S5_Figure. Spot dilution assay (10^6-10^1) demonstrates that *bta1*Δ and WT *C. neoformans* are similarly tolerant to stresses. Plates were prepared using YNB without phosphate as a base. Pi+ plates were supplemented with 29.4 mM KH_2PO_4, and Pi- plates were supplemented with 29.4 mM KCl. Strains were tested for their ability to grow at 37°C, in the presence of Amphotericin B and cell wall perturbing agents (Congo Red, Calcofluor White and SDS). To test the ability of the *bta1*Δ to assimilate carbon sources other than glucose, the glucose was replaced with lactate.
